# Relationship between expression mRNA gene Treg, Treg, CD4^+^, and CD8^+^ protein levels with TST in tuberculosis children: A nested case-control

**DOI:** 10.1016/j.amsu.2020.12.011

**Published:** 2020-12-19

**Authors:** Farsida, Rahmini Shabariah, Mochammad Hatta, Ilhamjaya Patellongi, Prihantono, Muh Nasrum Massi, Andi Asadul Islam, Rosdiana Natzir, Andi Dwi Bahagia Febriani, Firdaus Hamid, Fatimah, Risky Akaputra, Pitut Aprilia Savitri

**Affiliations:** a Faculty of Medicine and Health, Universitas Muhammadiyah Jakarta, Indonesia; b Faculty of Medicine, Universitas Hasanuddin Makassar, Indonesia

**Keywords:** Tuberculosis, Expression mRNA gene Treg, Treg, CD4^+^, CD8 +, Tuberculin skin test

## Abstract

**Background:**

The ability of *Mycobacterium tuberculosis* to survive intracellularly, provides a cellular adaptive immune response played by specific T cells to defend against tuberculosis. The adaptive immune response to Bacillus of Calmette and Guerin (BCG) immunization is responded to by B cells, T Follicular B helper, T regulatory, restriction CD1, CD8^+^, CD4^+^, Th1, Th2, and Th17. BCG immunization can cause a tuberculin test reaction to being positive. The tuberculin test is a method for diagnosing TB infection and for screening individuals for latent infection and assessing the rate of TB infection in a given population.

**Methods:**

a nested case-control survey was conducted on patients with a diagnosis of TB and parents 0–18 years of age from 3 hospitals in Indonesia during September–November 2019 with a total sample of 69 people undergoing clinical examinations, supporting and diagnosing subjects, blood sampling 1–2 cc for examination mRNA gene Treg, Treg, CD 4+, and CD 8+, then centrifuged at 3000 rpm for 10 min to support blood cells and serum.

**Results:**

There was a significant relationship between expression of mRNA gene Treg with TST (p = 0,000), Treg with TST (p = 0,000), and CD4^+^ with TST (p = 0,000). Meanwhile, CD8 + was not significantly associated with TST (p = 0.118).

**Conclusions:**

It is necessary to check the expression of mRNA gene Treg, Treg, CD4^+^, and CD8^+^ with more samples to find the mean value that shows the protective value of further TB.

## Highlights

• The Mantoux tuberculin skin test (TST) is one of the criteria to determine whether a person is infected with Mycobacterium tuberculosis. It is often used to screen individuals for latent infection and assess rates of TB infection in a given population. tuberculin test results with induration diameter> 10 mm were positive regardless of the cause. Patients in this study were children who had been vaccinated against BCG and diagnosed with TB in their early toddler years. It was necessary to see the adaptive immune response that existed when the child was infected with TB.
• The main immune response that acts as protection against TB is played by cellular immunity, namely CD4 and CD8 T cells. We thought that if we could find between TST and adaptive immune response, TST could be a prior test to determine prognosis or disease progression.
• We found a strong correlation between immune activation and Treg cell frequency in TST positive persons. A higher Treg consentration was found in TST positive subjects. Nevertheless, local immune responses may differ from those in peripheral blood and exploring this interaction in the site of active infection will give important clues about their involvement in protection or pathogenesis . According to our studies the increased Treg cell frequency observed in the blood of people with active tuberculosis is likely a response to inflammation and bacterial burden.

## 1 Introduction

Tuberculosis is caused by the bacteria *Mycobacterium tuberculosis* (Mtb), which is an infectious disease that can be treated, but the mortality rate caused by this disease is still high. In 2019 it is estimated that 10 million people suffer from tuberculosis worldwide, mostly in the Southeast Asia region, 44%. Indonesia is ranked second with the highest number of tuberculosis sufferers in Southeast Asia after India [1].

Based on Indonesia's health profile in 2019, the number of TB cases was 543,874 cases with the number of cases in men 1.4 times higher than women. The Case Detection Rate (CDR) of TB cases in Indonesia is 64.5% relatively higher compared to the previous 10 years. This figure is still far from the WHO recommendation, which is ≥ 90%. Provinces that have reached the CDR score are West Java and Gorontalo [2].

The protective effect provided by the BCG vaccine is mediated by innate immunity or the natural and adaptive immune system [3].

In adaptive immune response through CD4 + T cells as controlling the growth of Mtb germs and CD8 + T cells that produce gamma interferon (IFN-γ). Gamma interferon is one of the cytokines which plays a role in destroying the Mtb bacteria and interfering with the elimination process of the Mtb bacillus. The increase in IFN- γ production is expected to be able to protect the host when infected by the Mtb bacteria [4].

Another role in dealing with TB germs includes the T Regulator (Treg), which is a subset of CD4 + T cells. Treg cells function to suppress the CD4 + cell response efficiently. Tregs involved in controlling the immune response to self-Ags and in immune homeostasis are well known, and there is increasing evidence for a role for Tregs in regulating immunity to infection [5].

Tuberculin tests are performed on children who are suspected of being infected with TB, where this examination is one way to diagnose TB in children. Tuberculin is a pure protein obtained from TB bacteria (but does not contain active TB germs). The immunostimulatory ability of PPD protein with *Mycobacterium tuberculosis* is not much different. Tuberculin PPD RT23 was prepared in 1958 from several strains, including the strain of *M. tuberculosis* type H37RV isolated in 1905. Immunological as well as clinical responses between the two are highly relevant [6]. Most people living in countries with a high TB burden have latent TB infection. This condition is defined as a state of a persistent immune response to *Mycobacterium tuberculosis* (MTB) without the clinically manifested disease [7].

The tuberculin test can also be used to measure the prevalence of infection. From the prevalence of infection, it can be seen that the annual risk of tuberculosis infections (ARTI) by conversion method, and is one of the epidemiological parameters to determine the burden of disease (burden of tuberculosis). A study in Colombia in an area with a high TB case rate found 65% of tuberculin test results related to genome contribution. Specifically found in 128 families including 350 children who were included in the study showed a single 6000 nucleotide polymorphism identified at the major locus positive tuberculin test results on chromosome 11p14 region [8].

From the background above, the purpose of this study was to see the relationship between the role of adaptive immunity, namely expression mRNA gene Treg, Treg protein levels, CD4 +, and CD8 + to the Tuberculin Skin Test (TST) in tuberculosis children.

## 2 Methods

This study is an analytical study with a nested case-control design [9]. The study was conducted in 3 hospitals, namely the Jakarta Cempaka Putih Islamic Hospital, Central Jakarta, the Sukapura Islamic Hospital, North Jakarta, and the Bhakti Medicare Cicurug Hospital, Sukabumi Regency, West Java, which were conducted in September–November 2019. To the polyclinic of children diagnosed with TB and aged 0–18 years. The sample in this study was 69 people, the sample size was calculated using an unpaired numerical comparative formula for two groups of one measurement. The inclusion criteria in the study sample were (1) patients with intra- and extra-pulmonary tuberculosis determined by a pediatrician with experience in the hospital, (2) patients aged 0–18 years, (3) TB diagnosis for the first time was not relapsed or reinfection, and (4) have not taken OAT medication or the maximum if it has been taking OAT medication for less than 1 week.

Research data collection related to clinical examination, support, and diagnosis of the subject is carried out by a pediatrician separately, then the results are compiled in the patient's medical record. Taking 1–2 cc of blood samples for subjects who met the inclusion criteria for examination of mRNA gene Treg, Treg protein levels, CD 4 levels, and CD 8 levels, the samples were then centrifuged at 3000 rpm for 10 min to separate blood cells and serum. Blood serum was taken and put into an ependorphous tube and stored in a freezer/refrigerator at a temperature of −20 °C until all samples were filled. Besides, one drop of blood is included in a 500 μL aliquot.

The samples that met the requirements were brought to the Laboratory of Molecular Biology and Immunology Faculty of Medicine Universitas Hasanuddin in Makassar for the examination of the mRNA gene Treg, Treg protein levels, CD4^+^ levels, and serum CD8^+^ levels. The RNA extraction used the Guanidium thiocyanate and diatom method [10,11].

The mRNA concentration was measured with a QubitTM fluorometer (Invitrogen, Carlsbad, CA, United States) according to the manufacturer's 90 instructions, and the RNA samples were then stored at −80 °C until the next analysis. Examination of expression mRNA gene using the nucleic acid extraction method, expression mRNA gene Treg using the Real-Time Polymerase Chain Reaction (RT PCR) method, calculating the calibration curve with Ct (Cycle threshold) [[12], [13], [14], [15]].

Enzyme-linked Immunosorbent Assay (ELISA) to determine Treg, CD4 + and CD8^+^ serum levels was done according to protocol of previous study [[16], [17], [18]].

Data analysis in this study used univariate analysis to see the distribution of sample characteristics and bivariate analysis with an independent *t*-test to determine the difference in the mean of the two groups and their relationship. This study has received an ethical approval recommendation issued by the Health Research Ethics Committee of the Medical Faculty of Hasanuddin University Hospital on May 15, 2019, with the number 370/UN4.6.4.5.31/PP36/2019. This research in accordance with the Declaration of Helsinki with registration ID: researchregistry6236.

## 3 Results

From the existing samples, all 69 people have been given BCG vaccine. Table 1 shows the characteristics of the sample with the sex of male (52.5%) female (47.8%). Age 0–5 years (55.1%), 6–12 years (31.9%), 12–18 years (13%). TST negative (46.4%) and TST positive (53.6%), the average of expression mRNA gene Treg 10493.67 (2419,922), the average Treg protein content was 258402.55 (38032,582), CD4 + 63789.48 (16627,841), and CD8 + 63789.48 (16627.841).

**Table 1:** Sample characteristics (n = 69).

Measures	M (SD)	n (%)
Gender Men Women		36 (52.5) 33 (47.8)
Age 0–5 years 6–12 years 12–18 years		38 (55.1) 22 (31.9) 9 (13.0)
Tuberculin Skin Test Negative Positive		32 (46.4) 37 (53.6)
Expression mRNA Gene Treg	10493.67 (2419.922)	
Treg	258402.55 (38032.582)	
CD4^+^	63789.48 (16627.841)	
CD8^+^	515356.99 (105365.014)	

Table 2 shows a significant relationship between expression of mRNA gene Treg with TST (p = 0.000), Treg protein levels with TST (p = 0.000), and CD4 + with TST (p = 0.000). Meanwhile, CD8 + protein levels were not significantly associated with TST (p = 0.118).

**Table 2:** Relationship of expression mRNA gene Treg, Treg protein levels, CD4 +, and CD8 + with tuberculin skin test.

Measures	Tuberculin Skin Test	Mean	SD	SE	p-value
mRNA Gene Treg	Negative	9271.00	2152.114	380.444	0.000^a^
	Positive	11551.11	2143.596	352.405	
Treg	Negative	240990.50	33237.019	5875.530	0.000^a^
	Positive	273461.62	35754.694	5878.035	
CD4 +	Negative	55901.94	14469.801	2557.924	0.000^a^
	Positive	70611.14	15453.996	2540.621	
CD8 +	Negative	493980.13	93419.771	16514.438	0.118
	Positive	533845.08	112682.764	18524.932	

a Significant.

**Fig. 1:**
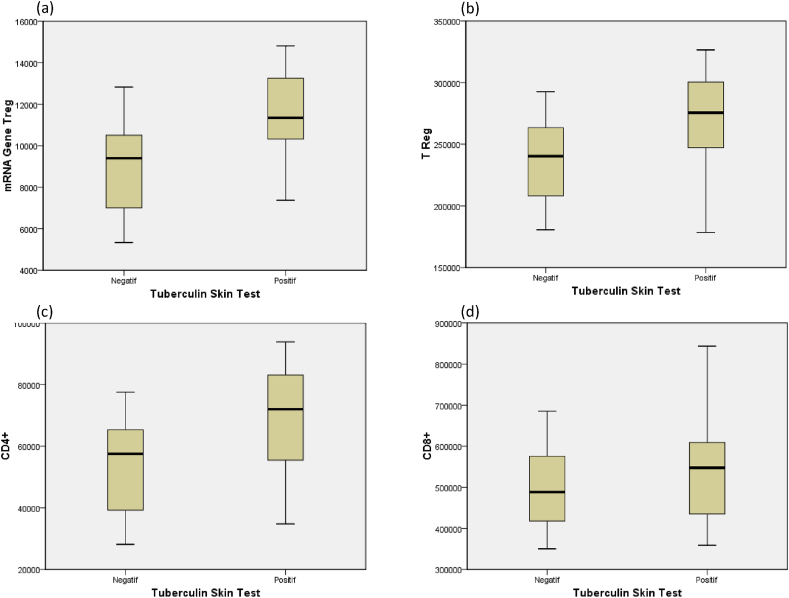
Expression mRNA gene Treg, Treg protein levels, CD4 +, and CD8 + on positive and negative tuberculin (TST) tests. (a) expression mRNA gene Treg according to TST. (b) Treg protein levels according to TST. (c) CD4 + according to TST. (d) CD8 + according to TST.

Fig. 1 showed a significantly higher expression mRNA gene Treg on positive TST (Fig. 1a). Treg protein levels were significantly higher in TST positive (Fig. 1b). CD4 + protein levels were significantly higher in TST positive (Fig. 1c). However, at CD8 + protein levels there was no significant difference between negative and positive TST (Fig. 1d).

## 4 Discussion

The Tuberculin Skin Test is important in identifying individuals with latent tuberculosis because in most individuals tuberculosis is initially controlled by the body's defenses and the infection remains latent. Identification and treatment of latent tuberculosis reduce the risk of developing active tuberculosis by as much as 90% among immunosuppressed individuals [19]. The results of this study obtained samples with a positive TST more than negative TST. In a study by Suvrat et al., it was found that two-thirds of TST-negative health workers showed positive results for T cell proliferation. In contrast, one-third of TST-positive subjects did not show an in vitro response. Similar in vivo and in vitro discordant responses have been previously reported as well. This suggests that people may exhibit unresponsiveness to TST even though they live in hyperendemic areas of TB. This is due to a lack of expression of ‘skin lymphocyte antigens' on skin-settling T cells [[20], [21], [22], [23]]. Deficiencies in the production of macrophage inhibiting factor (MIF) by skin macrophages can also contribute to poor TST response. The most likely reason behind the absence of a proliferative response in the T cell subset of the TST-positive group could be the result of the overproduction of TGF-β induced by PPD. As a ‘master regulator’ of the immune response, TGF-β can suppress the proliferative response of T cells [24].

In this study, TST was carried out on a sample of TB patients with more male sex than females. The sex differences in TB cases are related to hormonal and other risk factors such as smoking habits, which of course were not discussed in this study. Several studies suggest that smoking or having parents who smoke can increase TB cases in children. Other studies suggest that high and low levels of estrogen play an important role in the immune response to TB. Estrogen acts as an activator and promotes the action of Helper 2 T cells that induce the production of cytokines IL-4, IL5, and IL-1 [25]. The sample age was dominated by the 0–5 year age group and followed by the 6–12 year age group. This is in line with the research of Tori et al. By recording TB sufferers in children from 2007 to 2017, two-thirds of the patients were under 15 years old [26].

The analysis showed that the expression of mRNA gene Treg was significantly associated with TST. In a study comparing TB cases with infected and uninfected TB contacts as determined by TST and ELISPOT results, PBMCs from uninfected contacts had lower FOXP3 mRNA expression levels than TB cases. Contact with infected TB according to the authors may signal migration of Tregs to the lungs during initial infection, with reappearance in circulation during latent infection (established) [27].

This study also showed that Treg protein levels were significantly associated with TST, this is in line with the study conducted by Burl et al. which stated that the level of FOXP3 expression was significantly associated with newly infected contacts (TST +). Significant results have also been demonstrated in other studies conducted in HIV-infected individuals. A positive TST result can be seen from the increase in Treg cell activity. FOXP3, better known as Treg cells, is a factor related to the function of Treg cells. Although the various types of Tregs have been revealed by new studies to both occur naturally and be induced, FOXP3 is shown to have a direct role in inducing immunosuppression [[27], [28], [29]].

In this study, CD4 + was significantly associated with TST, this is in line with the study of Rodriguez et al., which stated that people with positive tuberculin cells had higher CD4 + counts than tuberculin negative people [30]. However, these results are not in line with a study conducted by Sarrazin et al. which found that CD4 + cell count was not associated with TST [28]. In another study, there was a positive correlation between the CD4 + T lymphocyte count and the TST measurement value (r = 0.3, p = 0.003). As the number of CD4 + T lymphocytes increases, TST positivity increases (p = 0,007) [31].

In another study, CD4 T cells in the TST positive group had a more reactive response to PPD. This can be seen from the surface molecules that are mostly positive for CD45RO and CD127, and negative for CD27, CCR7, CD62L, and CD38. This suggests that PPD-reactive CD4 T-cells are end-differentiated effector memory T-cells that have lost the ability to circulate to lymphatic tissue. Cytokine profiles show strong expression of tumor necrosis factor (TNF) -α, and IFN-γ. Meanwhile, IL-4, IL-5, and IL -17 could not be detected. Also, no PD-1 and CTLA-4 were found, indicating the functional integrity of specific T-cells [20].

A weakened immune system and a higher risk of opportunistic infections can be seen in people who have low CD4 + levels. CD4 + values in the immune system of normal people are generally between 1400 and 1500. This number can decrease continuously in people who have low immunity. One example is people who are infected with HIV, in many cases people with HIV even have CD4 + levels reaching zero.

In this study, it showed that CD8 + was not significantly related to TST, this is in line with the study of Rodriguez et al. in HIV positive people, it was found that there was no difference in CD8 + counts in positive and negative tuberculin counts. In other studies, it was also found that there was no correlation between TST + and CD8 + [22,30]. CD8 + or cytotoxic T cells are the destroyer of virus-infected cells, tumor cells and are involved in organ transplant rejection. They are called CD8 + T cells because there are CD8 + glycoproteins on the cell surface that bind to MHC class I. In autoimmune disease, cytotoxic T cells can become passive in anergic status [32]. These cells can also be activated directly by recognizing TB infected cells. CD8 + T cells play a role in controlling the replication of Mtb bacteria by producing IFN-γ. These cells can also mediate the killing process of macrophages infected with Mtb [33].

## 5 Conclusion

In conclusion, this study, conducted against the backdrop of a persistent TB epidemic, requires a serious assessment of the methods and protocols currently used for TB prevention and detection. Examination of expression mRNA gene Treg, Treg protein levels, CD4 + and CD8 + with more samples to find the mean value that shows the protective value of further TB.

## Declarations

The authors declare that there are no competing interests associated with the manuscript. This research is fully funded by Faculty of Medicine and Health, Universitas Muhammadiyah Jakarta.

Provenance and peer review.

Not commissioned, externally peer reviewed.

## Declaration of competing interest

We wish to confirm that there are no known conflicts of interest associated with this publication and there has been no significant financial support for this work that could have influenced its outcome.
